# Peri-Ureteral Abscess Formation Following Ureteroscopic Laser Lithotripsy: A Case Report

**DOI:** 10.7759/cureus.29165

**Published:** 2022-09-14

**Authors:** Erik Jon Hammond, Vincent Grekoski, Amit Boukai, Glenn Goodwin, Laurence Dubensky

**Affiliations:** 1 Emergency Medicine, Aventura Hospital and Medical Center, Miami, USA; 2 Osteopathic Medicine, Touro College of Osteopathic Medicine, New York, USA

**Keywords:** percutaneous drainage peri-ureteral abscess, periureteral abscess and dvt, post-ureteral stent abscess, genitourinary abscess, ureteral stent complication, urologic abscess, peri-ureteral abscess

## Abstract

We report the case of a patient with a previous history of obstructive renal calculus disease who initially presented with a symptomatic calculus in her right mid-ureter, requiring ureteroscopy with laser lithotripsy and ureteral stent placement. Shortly after the removal of the stent, the patient was found to have a peri-ureteral abscess, necessitating percutaneous drainage by interventional radiology, and placement of an additional ureteral stent. Adverse reactions to these procedures are rare and, to our knowledge, this is the only documented case of peri-ureteral abscess as a complication of ureteroscopic laser lithotripsy or of ureteral stenting. In addition to developing a peri-ureteral abscess, this patient also experienced deep vein thrombosis (DVT) and subsegmental pulmonary embolism (PE), which also have not been found to be a common complication of laser lithotripsy or ureteral stent placement in any of the studies that we reviewed for this article. The complications that were previously rare are unfortunately on the rise, possibly in the setting of both increased access to invasive therapies as well as the increased rates of diabetes and obesity. Survivability hinges on prompt recognition and treatment of these complications. In the event that a peri-ureteral abscess is discovered, prompt treatment with broad-spectrum antibiotics is recommended in addition to interventional radiology and urology consultation. Antibiotics should cover conventional intra-abdominal and urologic abscess regimens.

## Introduction

The two most common methods for stone extraction are shockwave lithotripsy (SWL) and ureteroscopic (URS) laser lithotripsy. While SWL employs sound waves to break up the stone and does not require an endoscope, URS laser lithotripsy requires a scope, high-frequency laser, and sometimes a stone basket, and/or grasping forceps [1,2]. Additionally, URS procedures are typically followed by ureteral stent placement in order to optimize post-procedure course and outcomes [2]. Interestingly, while URS utilizes more tools and steps, it generally has a lower rate of complications than SWL, although both are considered very low-risk procedures [3,4]. To our knowledge, the case we present here is the only case of abscess formation in the setting of URS. Our patient was also found to have deep vein thrombosis (DVT) and subsegmental pulmonary embolism (PE) formations during her hospital course, neither of which had previously been associated with any of the aforementioned urological procedures. We present this rare case and also explore various possibilities to account for the complications.

## Case presentation

**Figure 1:**
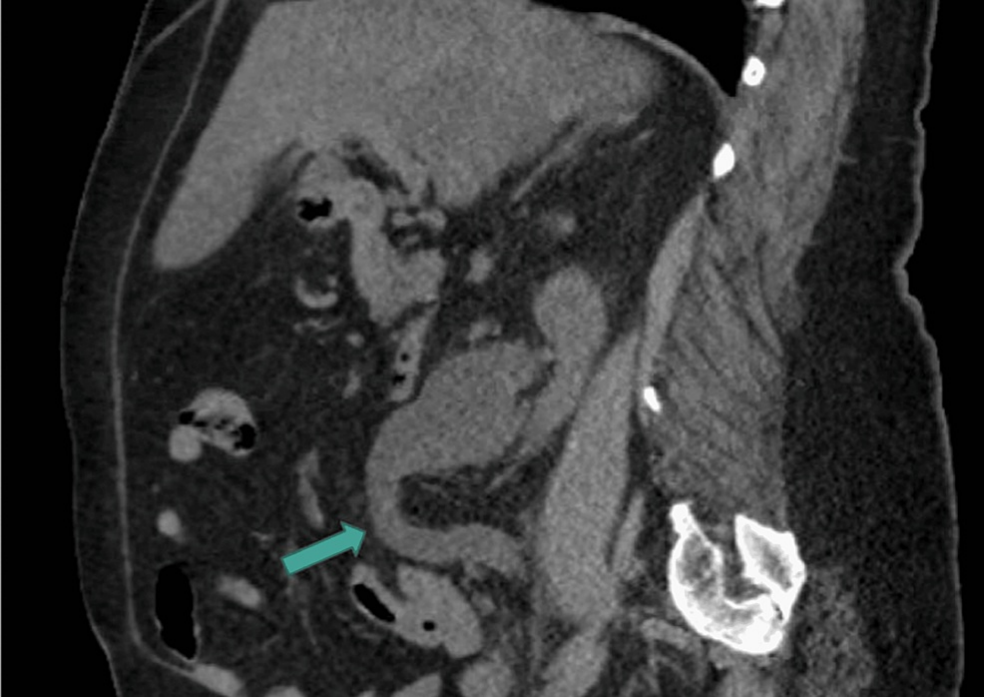
CT sagittal view revealing hydronephroureter CT: computed tomography

**Figure 2:**
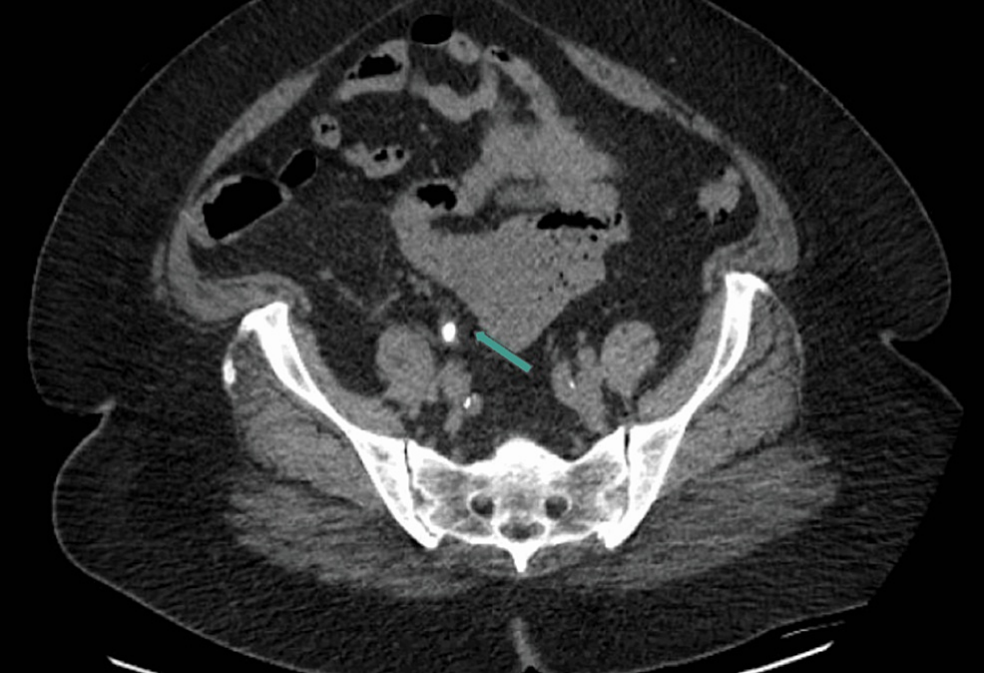
CT transverse view demonstrating obstructive ureteral calculi CT: computed tomography

A 58-year-old Caucasian female presented to the ED with a three-day history of right-sided flank pain, fever, and nausea. She was initially afebrile, with vital signs significant only for tachycardia of 117 beats per minute (BPM). Her physical exam revealed normal cardiac and pulmonary parameters, with the exception of tachycardia; the exam revealed no rash or neurologic deficits but elicited tenderness to percussion in the right flank. Laboratory analysis revealed a creatinine of 1.5 mg/dL (increased from a baseline of 1.0 mg/dL), blood urea nitrogen (BUN) of 24 mg/dL, hemoglobin of 10.1 g/dL, and white count of 8.0 x 10^9^/L. Her urinalysis was significant for 100 mg protein, >20 red blood cells (RBCs), >100 leukocytes (WBCs), positive leukocyte esterase, and "many" bacteria. She underwent CT cross-sectional imaging of her abdomen and pelvis, which revealed multiple mid-ureteral calculi measuring up to 6 mm, resulting in severe proximal right hydronephroureter as demonstrated in Figure 1 and Figure 2.

She was admitted to the inpatient medicine service for the management of obstructive ureteral calculi. After evaluation by the urology service, the patient underwent cystoscopy, right ureteroscopy with laser lithotripsy, and ureteral stent placement. The remainder of her hospital course was uneventful and she was discharged on day five with a 10-day course of ampicillin 500 mg every six hours (q6h) and ciprofloxacin 500 mg q12h. 

**Figure 3:**
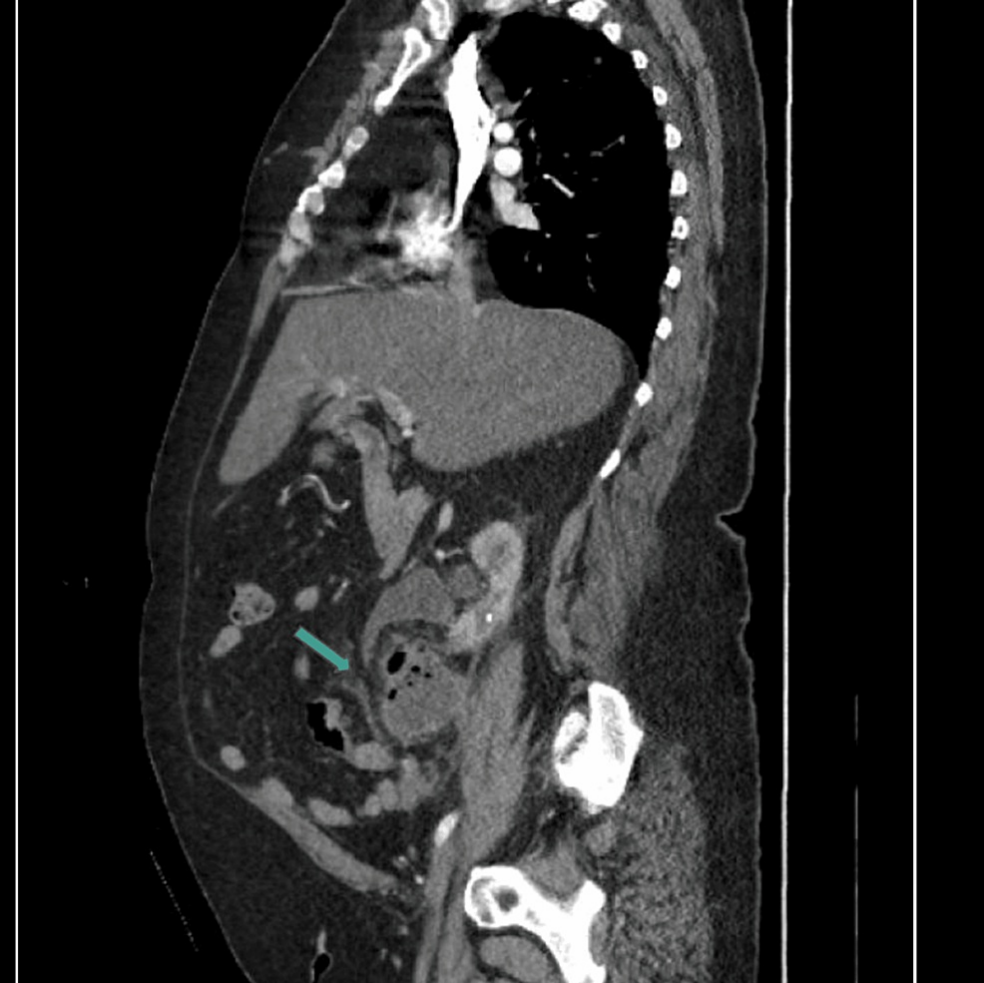
CT sagittal view representing the peri-ureteral fluid and gas-filled lesion, consistent with abscess CT: computed tomography

**Figure 4:**
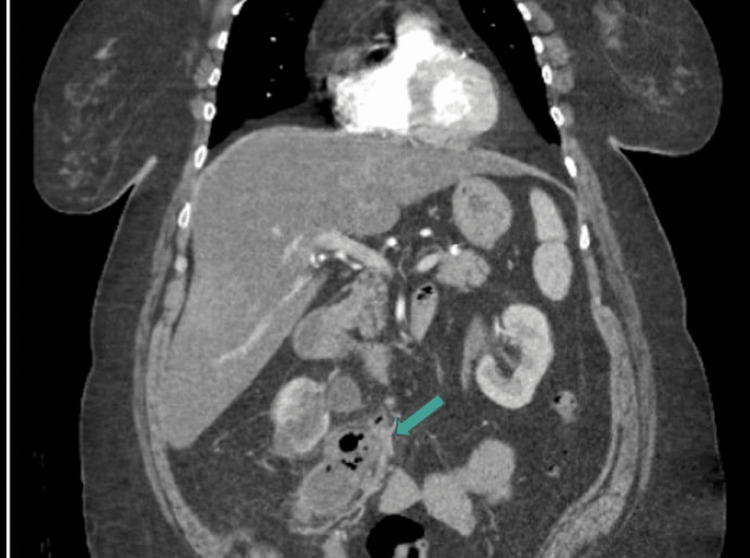
CT coronal view of gas and fluid-filled peri-ureteral structure, consistent with abscess CT: computed tomography

**Figure 5:**
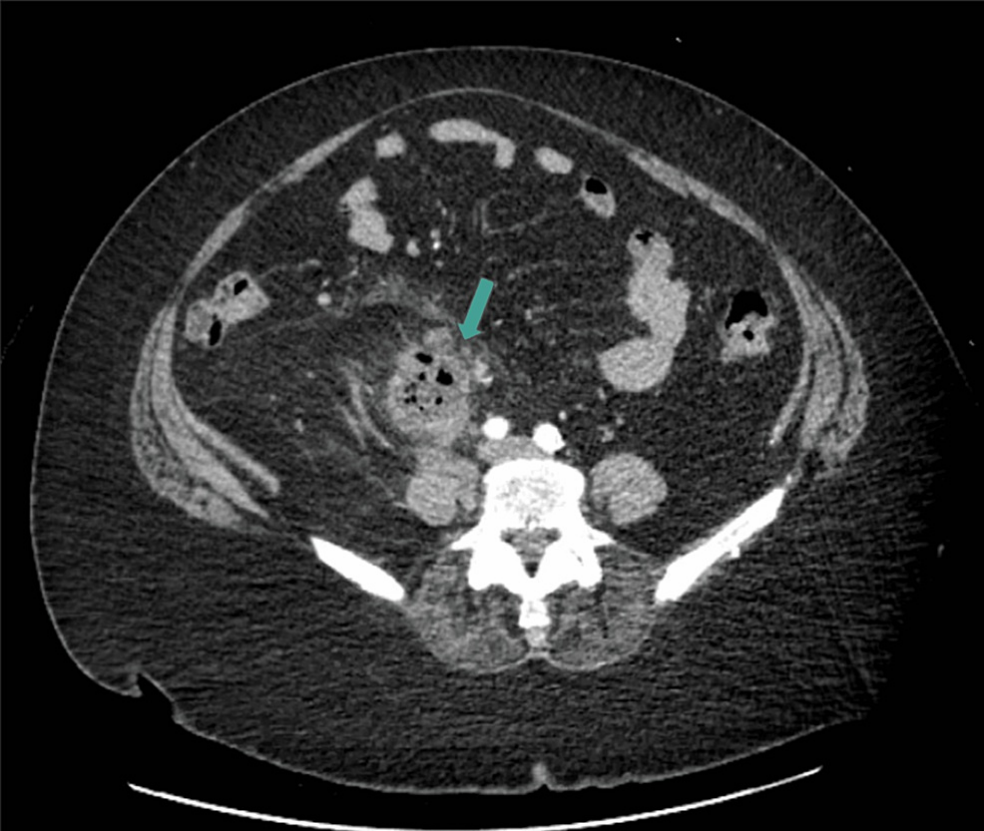
Cross-sectional CT transverse view demonstrating a gas- and fluid-filled peri-ureteral structure, consistent with abscess CT: computed tomography

The patient subsequently returned to the ED approximately 15 days after discharge with complaints of acute abdominal pain, shortness of breath, nausea, vomiting, and diarrhea. Three days prior to the presentation, she had the aforementioned ureteral stent removed by her urologist as an outpatient. On presentation, she was afebrile, with vital signs significant only for tachycardia of 126 BPM. Her physical exam revealed a firm, distended, and diffusely tender abdomen, most notable in the epigastric region. Laboratory analysis revealed a creatinine of 1.3 mg/dL, BUN of 22 mg/dL, hemoglobin of 9.1 g/dL, and white blood cell count of 8.9 x 10^9^/L. Her urinalysis was significant for 30 mg protein, <20 RBCs, >100 WBCs, positive leukocyte esterase, and rare bacteria. Due to the nature of her symptoms in the setting of a recent hospitalization and procedure, she underwent both CT angiography of the chest and CT cross-sectional imaging of her abdomen and pelvis with contrast, which revealed a subsegmental PE, an abscess located adjacent to the right mid-ureter with gas, and a distal right ureteral stone causing mild hydronephrosis. The abdominal CTs revealing the abscess are shown in Figures 3-5.

The patient was admitted to the inpatient medicine service for the management of peri-ureteral abscess, ureteral calculus, and subsegmental pulmonary embolus.

She was evaluated by the interventional radiology team who proceeded with CT-guided aspiration and drain placement of the right retroperitoneal fluid collection with the placement of an inferior vena cava (IVC) filter. In addition, she had a percutaneous nephrostomy catheter placed due to significant right-sided hydronephrosis. On admission, she was empirically started on piperacillin-tazobactam. After significant resolution of her retroperitoneal abscess and hydronephrosis, both drains were removed. Urology proceeded with cystoscopy, right ureteroscopy with basket extraction of the stone, and ureteral stent placement. The subsequent ureteroscopy revealed the resolution of the abscess without signs of perforation or false lumens.

The remainder of the patient’s course was unremarkable and she was discharged home on hospital day 13 with a 15-day course of amoxicillin-potassium clavulanate 875-125 mg q12h.

## Discussion

While the vast majority of genitourinary stone patients experience spontaneous resolution, a significant number of patients require intervention. Some of the more common surgical interventions are SWL and URS laser lithotripsy. The basic mechanism of SWL involves high-energy shock waves produced by an electrical discharge [1]. The shock waves are transmitted through water and directly focused onto a kidney/ureteral stone with the aid of biplanar fluoroscopy [1]. The change in tissue density between the soft kidney tissue and the hard stone causes a release of energy at the stone surface, usually resulting in fragmentation [1]. This is in contrast to the more precise procedure of URS, where a small endoscope (either rigid, semirigid, or flexible) is passed from the urethra proximally toward the affected ureter and kidney [2]. Once the stone is visualized, it can either be extracted by stone baskets or grasping forceps or can be broken up using laser lithotripsy [2]. Because of the instrumentation needed for URS, ureteral dilation is often required prior to endoscopic insertion [2]. With the development of smaller-caliber semirigid and flexible ureteroscopes and the introduction of improved instrumentation, including the holmium:yttrium-aluminum-garnet (YAG) laser, URS has evolved into a safer and more efficacious modality for the treatment of ureteral stones in all locations [3,4].

Ureteroscopic lithotripsy is often followed by ureteral stent placement in order to aid with the relief of postoperative pain, prevent ureteral obstruction, improve hydronephrosis, improve healing, and prevent ureteral stricture [5,6]. It is one of the safest forms of treatment for urolithiasis but may cause urothelial tissue injury adjacent to the targeted stone, sometimes progressing to stricture development [7,8]. The only contraindication for laser lithotripsy is the presence of an untreated urinary tract infection (UTI) due to the risk of urosepsis [8]. Life-threatening complications of ureteroscopy include renal rupture, perirenal hematoma, subcapsular hematoma, acute sepsis, arteriovenous (AV) fistula formation, ureter avulsion, and retroperitoneal hematoma [9]. Other complications that have been observed include lost stone, ureteral perforation/extravasation/avulsion, or even fatal gas embolism [8]. One study analyzed 433 adverse events caused by different forms of lasers used in urology [10]. There were seven reported deaths, four of which were caused by air emboli and the remaining three were due to ureteral perforation with resulting retroperitoneal bleeding [10]. Additionally, eight cases experienced major complications requiring open surgery for the management of ureteral perforation, ureteral avulsion, bladder perforation, or broken equipment requiring retrieval [10]. However, the study acknowledges that these complications were voluntarily reported, thereby constituting a considerable limitation [10].

In addition to the aforementioned mechanical complications of ureteroscopy, lithotripsy, and stent placement, there are some additional infectious complications. The placement of ureteral stents has been associated with the formation of biofilms, resulting in complicated UTIs [11]. Biofilms are more commonly seen in patients with diabetes and chronic kidney disease [11]. Along with biofilm formation, complications of abscess formation can also occur, which are exceedingly rare. Only a few case studies have reported post-lithotripsy/stent abscess formation and these were retroperitoneal [12-14]. While there have been cases of renal abscess formation, to our knowledge, this is the only documented case of a peri-ureteral abscess occurring as a complication of ureteroscopic laser lithotripsy or of ureteral stenting.

In addition to developing a peri-ureteral abscess, our patient also experienced DVT and subsegmental PE, which also have not been found to be a common complication of laser lithotripsy or ureteral stent placement in any of the studies that we reviewed for this article. Although it is worth noting that the DVT and PE may have been more related to the patient's positioning and decreased activity more than the actual procedure itself. In high-risk patients in need of post-procedure hospitalization, current international guidelines recommend systemic anticoagulation therapy with low-molecular-weight heparin (LMWH) as a subcutaneous injection once daily for the duration of hospitalization or until complete mobility is possible [15]. In a meta-analysis of 1,109 patients on anticoagulation who underwent ureteroscopic intervention for the treatment of nephrolithiasis, there was only one case of DVT in the bleeding diathesis group and one in the control arm, demonstrating a low risk of thrombosis regardless of anticoagulation status [16]. This low prevalence rate further highlights the unique presentation of the patient in our case study. The only case studies published associating PE with these procedures involved calcular PE resulting from renal stone fragments or gas entering into the venous circulation [17,18].

## Conclusions

This case and background evidence highlight the disturbing trend of previously rare complications being on the rise. Many factors could be responsible for this trend, including increased access to invasive therapies as well as the persistently high rates of diabetes and obesity, as epitomized by our patient. This makes it increasingly more difficult for the ED physician, considering the efforts to be more judicious with imaging and broad-spectrum antibiotics. The aforementioned case serves as a reminder for ED physicians to be aware of these rare complications while also highlighting the need to conduct more research, and develop more comprehensive management algorithms. Based on the progression of this case as well as an analysis of the limited evidence, patients presenting with post-genitourinary procedure abscesses should be promptly treated with broad-spectrum antibiotics in addition to interventional radiology and urology consultation. Antibiotics should cover conventional intra-abdominal and urologic abscess regimens.
